# Use of count-based image reconstruction to evaluate the variability and repeatability of measured standardised uptake values

**DOI:** 10.1371/journal.pone.0192549

**Published:** 2018-02-12

**Authors:** Tomohiro Kaneta, Hiromitsu Daisaki, Matsuyoshi Ogawa, En-Tao Liu, Hitoshi Iizuka, Tetsu Arisawa, Ayako Hino-Shishikura, Keisuke Yoshida, Tomio Inoue

**Affiliations:** 1 Department of Radiology, Yokohama City University, Yokohama, Japan; 2 Department of Radiological Technology, Gunma Prefectual College of Health Sciences, Maebashi, Japan; Biomedical Research Foundation, UNITED STATES

## Abstract

Standardized uptake values (SUVs) are the most widely used quantitative imaging biomarkers in PET. It is important to evaluate the variability and repeatability of measured SUVs. Phantom studies seem to be essential for this purpose; however, repetitive phantom scanning is not recommended due to the decay of radioactivity. In this study, we performed count-based image reconstruction to avoid the influence of decay using two different PET/CT scanners. By adjusting the ratio of ^18^F-fluorodeoxyglucose solution to tap water, a NEMA IEC body phantom was set for SUVs of 4.0 inside six hot spheres. The PET data were obtained using two scanners (*Aquiduo* and *Celesteion;* Toshiba Medical Systems, Tochigi, Japan). We set the start time for image reconstruction when the total radioactivity in the phantom was 2.53 kBq/cc, and employed the counts of the first 2-min acquisition as the standard. To maintain the number of counts for each image, we set the acquisition time for image reconstruction depending on the decay of radioactivity. We obtained 50 images, and calculated the SUV_max_ and SUV_peak_ of all six spheres in each image. The average values of the SUV_max_ were used to calculate the recovery coefficients to compare those measured by the two different scanners. Bland-Altman analyses of the SUVs measured by the two scanners were also performed. The measured SUVs using the two scanners exhibited a 10–30% difference, and the standard deviation (SD) of the measured SUVs was between 0.1–0.2. The *Celesteion* always exhibited higher values than the *Aquiduo*. The smaller sphere exhibited a larger SD, and the SUV_peak_ had a smaller SD than the SUV_max_. The Bland-Altman analyses showed poor agreement between the SUVs measured by the two scanners. The recovery coefficient curves obtained from the two scanners were considerably different. The *Celesteion* exhibited higher recovery coefficients than the *Aquiduo*, especially at approximately 20-mm-diameter. Additionally, the curves were lower than those calculated from the standard 30-min acquisition images. We propound count-based image reconstruction to evaluate the variability and repeatability of measured SUVs. These results are also applicable for the standardization and harmonization of SUVs in multi-institutional studies.

## Introduction

The standardized uptake value (SUV) is the most widely used quantitative imaging biomarker (QIB) in the field of positron emission tomography (PET). This quantitative value has been used to determine and evaluate differential diagnoses, therapeutic effects, and prognostic predictions [1–5]. It is well known that SUVs are influenced by many factors, such as the type of scanner and workstation, imaging protocol, body habitus, disease distribution, image noise, and radioactivity [6–8]. Recently, the Radiological Society of North America (RSNA) organized a Quantitative Imaging Biomarkers Alliance (QIBA) to improve the value and practicability of quantitative imaging biomarkers by reducing variability across devices, patients, and time [9]. To evaluate the variability and repeatability of measured SUVs, phantom studies seem to be essential. However, a standard method to evaluate them has yet to be established. One of the simplest methods may be repetitive scanning of a phantom. However, this method does not consider maintaining the condition of the phantom, even if repetitive scanning of the same phantom is performed continuously. The measured SUV will increase over time due to the increase of noise from the decay of radioactivity [10]. Thus, it is essential to design a method of repetitive phantom scanning that avoids the influence of radioactive decay. We therefore developed a method that maintains the total count for each image, by setting the time and duration for image reconstruction. In the current study, we performed this count-based image reconstruction (using a fixed average number of counts per image) instead of time-based image reconstruction (using a fixed acquisition time per image), and evaluated the variability and repeatability of measured SUVs using two different PET/computed tomography (CT) scanners.

## Methods

### Phantom preparation

An image-quality, International Electrotechnical Commission (IEC) body phantom of the type described in the National Electrical Manufacturers Association (NEMA) NU-2 2012 Standard [11] was used for the experiments. First, we measured the background volume of the phantoms. Then, using a regularly checked dose calibrator and taking decay into consideration, ^18^F-fluorodeoxyglucose (FDG) solution was prepared and measured. Next, exactly one-fourth of the background volume was filled with tap water, and a precise amount of ^18^F-FDG was added to produce a hot solution. An aliquot of this solution was added into all six (10-, 13-, 17-, 22-, 28-, and 37-mm-diameter) hot spheres. The phantom background was filled with tap water and stirred to produce a warm solution. The resultant SUV of each sphere was 4.0. The radioactivity of the ^18^F-FDG used for the experiments was measured and corrected using a standard radiation source (^68^Ge Dose Calibrator Source [37 MBq] BM06S-CE; RadQual Global Sources, NH, USA).

### PET/CT scan

We used two different PET/CT scanners, developed by Toshiba Medical Systems (*Aquiduo* and *Celesteion*; Tochigi, Japan); both scanners combine a 16-multislice CT scanner with a high-resolution PET scanner. The *Aquiduo* scanner was released in 2005, and uses lutetium oxyorthosilicate (LSO) as detector material [12]. The *Celesteion* scanner was released in 2014, and uses lutetium-yttrium oxyorthosilicate (LYSO) with a time-of-flight (TOF) function. The TOF temporal resolution was reported to be < 450 ps by Toshiba [13].

A CT transmission scan was performed using a 120-kV tube voltage. The field-of-view (FOV) was set at 500 mm for the *Aquiduo* scanner, and 550 mm for the *Celesteion* scanner. Subsequently, two consecutive 2-h PET emission scans were performed using list-mode (total, 4 h). Two hours is the maximum duration of continuous scanning for these scanners.

For reconstruction, a two-dimensional (2D) ordered subset expectation maximization (OSEM) algorithm was used for the *Aquiduo* scanner, and a three-dimensional (3D) OSEM algorithm was used for the *Celesteion*. The TOF function was used for the *Celesteion*. The pixel size was 2 mm for both scanners, and post-reconstruction Gaussian filtering was applied with a 6-mm full-width at half-maximum (FWHM) for the *Aquiduo* scanner, and a 7-mm FWHM for the *Celesteion*. These are the protocols used for daily clinical PET examinations in our hospital.

### Count-based reconstruction

To maintain the number of counts for each image, we set the start time and duration for image reconstruction depending on the decay of radioactivity. In Japan, 3.7 kBq/kg of ^18^F-FDG is commonly administered one hour prior to PET scan commencement. This corresponds to 2.53 kBq/cc at the beginning of the scan. We set the start time for image reconstruction when the total radioactivity in the phantom was 2.53 kBq/cc, and employed the counts of the first image reconstruction as the standard. Considering the measured radioactivity in the phantom and the decay of ^18^F-FDG (109 min), the start time and duration for image reconstruction were determined to make the total counts in each reconstruction match (Table 1).

**Table 1:** Time calculations for image reconstruction.

Reconstruction No.	Time (min:sec)	Duration (min:sec)	Decay factor
1	(A)	2:00	1
2	(A) + 2:00	2:02	0.987
3	(A) + 4:02	2:03	0.975
4	(A) + 6:05	2:05	0.962
5	(A) + 8:10	2:06	0.950
6	(A) + 10:16	2:08	0.937

At the time (A), the total radioactivity in the phantom corresponds to 2.53 kBq/cc

Decay factor = $e^{-\frac{elapsed\;time\;after(A)}{half\;life}\times ln(2)}$

Duration = 2 min / Decay factor, e.g., Duration for reconstruction no. 2 = 2 min / 0.987

For example, for the first image reconstruction, the data were obtained between (A) and (A) + 02:00 (acquisition time: 2 min); the second reconstruction was obtained between (A) + 02:00 and (A) + 04:02 (2 min 2 sec), and the third between (A) + 04:02 and (A) + 06:05 (2 min 3 sec), where (A) was the time when the total radioactivity in the phantom corresponded to 2.53 kBq/cc. Fifty images were reconstructed for each scanner. For comparison, we also reconstructed images every 2 min.

### SUV measurements

The SUV_max_ and SUV_peak_ of all six spheres in the 50 reconstructed images were calculated using GI-PET (AZE Co. Ltd., Tokyo, Japan). The SUV_peak_ was defined as the average SUV within a small sphere (12-mm-diameter) centred on the highest uptake region in the volume of interest (VOI), in contrast to the SUV_max_, which was defined as the most intense voxel within the VOI. Both the SUV_max_ and SUV_peak_ of all six spheres (inner diameters of 37, 28, 22, 17, 13, and 10 mm) in the phantom were measured using a VOI that covered the entire sphere, and recorded for all reconstructed images.

### Recovery coefficient

The recovery coefficient (RC) for a *j*-mm-diameter hot sphere (RC_*j*_) is the quotient of the maximum pixel value (C_*j*_) within the region of interest over the sphere on the reconstructed image and the maximum pixel value of a 37-mm-diameter sphere (C_37_): RCj=CjC37. These coefficients are calculated using the averaged values obtained from all 50 images. Additionally, the values obtained from the image corresponding to the 30-min acquisition were also calculated as the standard RCs.

### Repeatability

To evaluate the repeatability of the SUVs measured by the two scanners, Bland-Altman plot analyses were performed.

## Results

**Fig 1:**
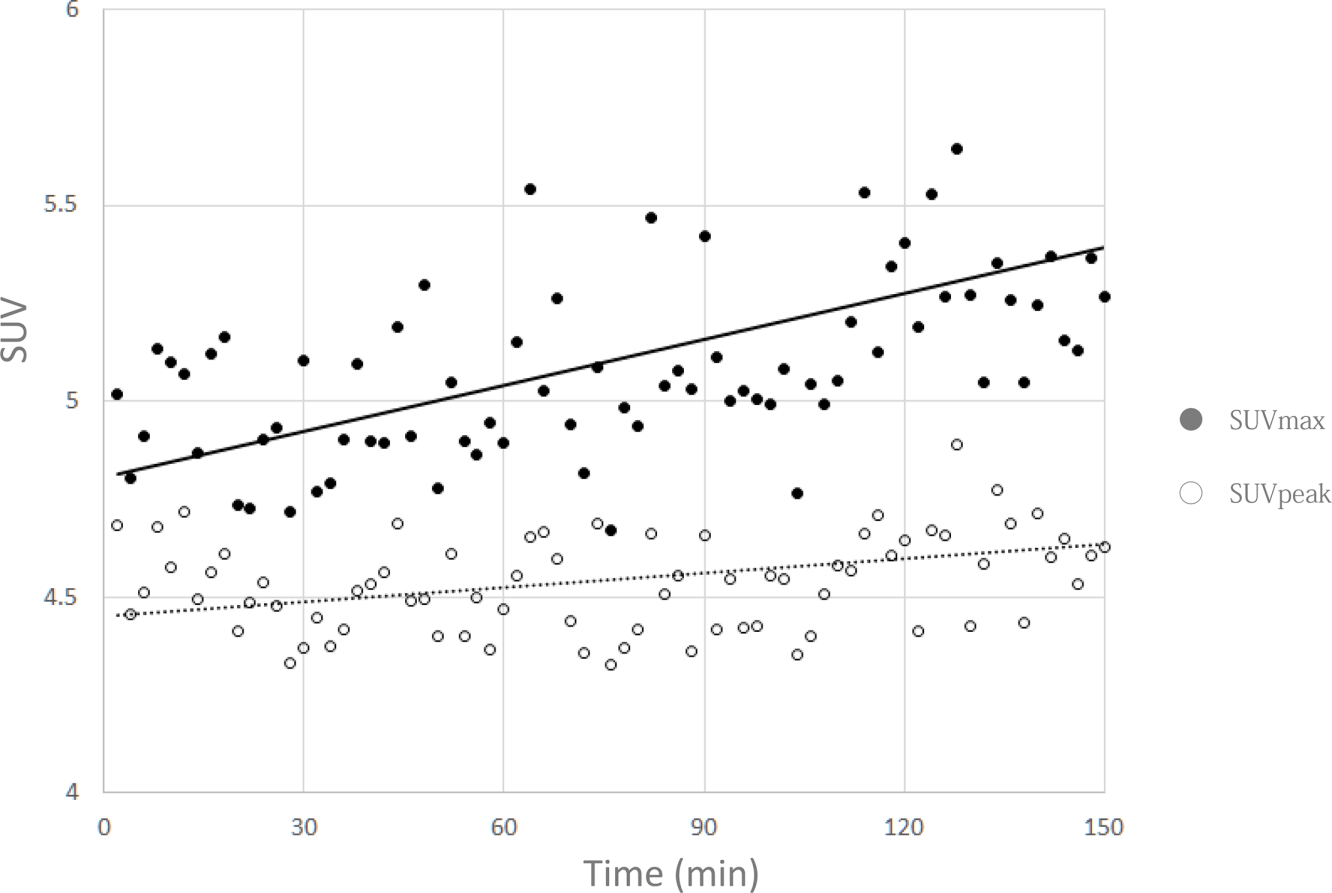
The measured SUV_max_ and SUV_peak_ of a 37-mm-diameter sphere with a 2-min acquisition over time. Standardized uptake values (SUVs) obtained from constant 2-min acquisition periods using the *Celesteion* scanner are shown over time to compare the resulting SUVs. The scatter plot depicts SUV_max_ (solid, black circle) and SUV_peak_ (empty/open circle) values. The solid and dotted trend lines correspond to SUV_max_ and SUV_peak_, respectively.

The total radioactivity of the ^18^F-FDG solution at the time of phantom preparation was 37.9 MBq (3.84 kBq/cc) for the *Celesteion* scanner, and 27.3 MBq (2.77 kBq/cc) for the *Aquiduo*. As mentioned in the Methods section, we set the start of image reconstruction at the time when the total radioactivity in the phantom was 2.53 kBq/cc, and used the counts for the first 2-min acquisition period as the standard. Before we demonstrate the results of our count-based reconstruction method, we show the trend of measured SUVs obtained with a constant acquisition period in Fig 1. The SUV_max_ and SUV_peak_ of the 37-mm-diameter sphere are shown for constant 2-min acquisition periods using the *Celesteion* scanner. The x-axis represents the time after scanning commenced. The SUVs tended to increase, especially the SUV_max_. The SUV_max_ also exhibited a gradual increase in variation over time. Results obtained using the *Aquiduo* are omitted.

### Variations of measured SUVs

**Fig 2:**
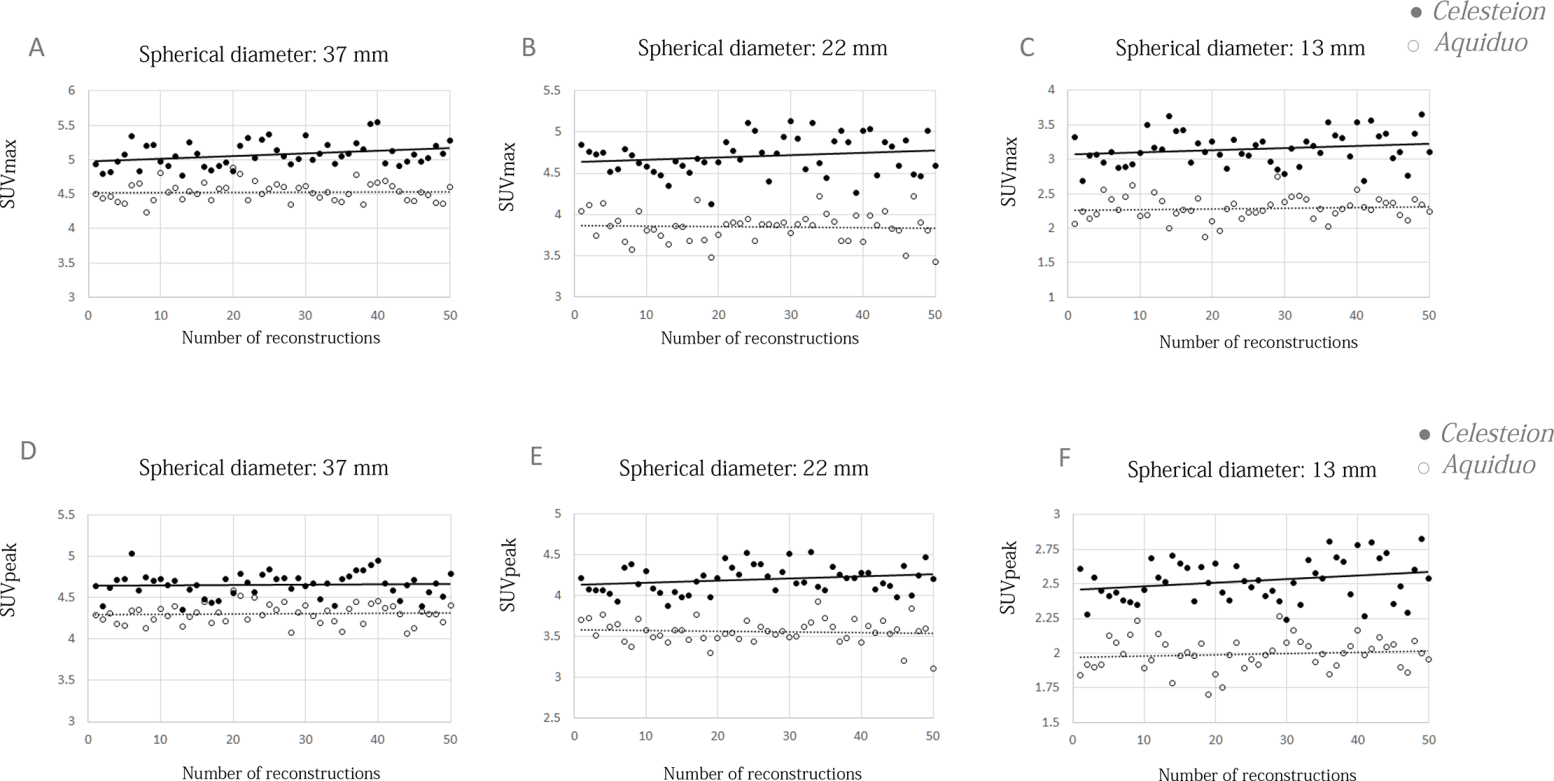
The distributions of count-based measured SUV_max_ and SUV_peak_. The distribution of the SUV_max_ (A-C) and SUV_peak_ (D-F) of the representative three spheres. The 50 count-based measurements were performed using the *Celesteion* (solid, black circle) and *Aquiduo* (empty/open circle) scanners. The solid and dotted trend lines correspond to the *Celesteion* and *Aquiduo*, respectively.

Fig 2 shows the distributions of the SUV_max_ and SUV_peak_, respectively, of three of the six spheres (inner diameter of Sphere; 37 mm, 22 mm and 13 mm). The increasing tendencies of the values and variations were not obvious for both SUV_max_ and SUV_peak_.

Table 2 summarises the mean and standard deviation (SD) values of the measured SUVs.

**Table 2:** Mean and standard deviation (SD) of measured SUVs.

Diameter of the sphere	37mm	22mm	13mm
*Celesteion*			
SUV_max_ Mean	5.077	4.707	3.150
SD	0.146	0.189	0.194
SUV_peak_ Mean	4.657	4.194	2.523
SD	0.112	0.136	0.125
*Aquiduo*			
SUV_max_ Mean	4.534 (89.3%)	3.848 (81.8%)	2.291 (72.7%)
SD	0.110	0.135	0.132
SUV_peak_ Mean	4.303 (92.3%)	3.560 (84.9%)	1.994 (79.0%)
SD	0.095	0.109	0.090

The percentage in parentheses represents the ratio to the SUVs measured by *Celesteion*.

### Repeatability of SUVs

**Fig 3:**
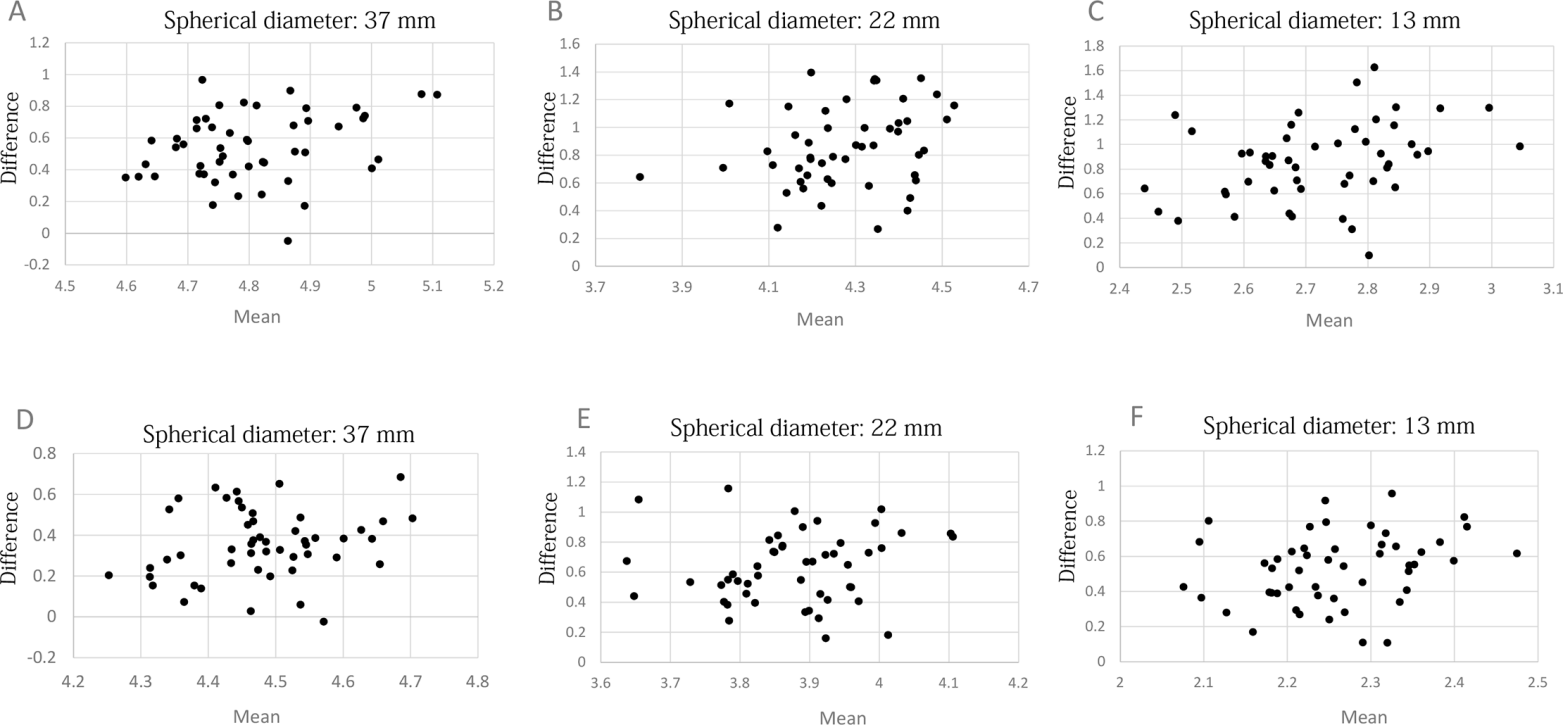
The Bland-Altman plots of count-based measured SUV_max_ and SUV_peak_. The Bland-Altman plots of SUV_max_ (A-C) and SUV_peak_ (D-F) measured by the *Celesteion* and *Aquiduo*.

Fig 3 depicts the Bland-Altman plots of the SUVs for three of the six spheres measured by the *Celesteion* and *Aquiduo* scanners.

The mean, SD, and 95% confidence interval (CI) values are shown in Table 3.

**Table 3:** The results of Bland-Altman analyses of the SUVs measured by the *Celesteion* and *Aquiduo* scanners.

Diameter of the sphere (mm)	37	28	22	17	13	10
SUV_max_						
Difference Mean	0.543	0.587	0.859	0.969	0.859	0.590
SD	0.177	0.196	0.237	0.253	0.251	0.215
95% CI	0.049	0.054	0.066	0.070	0.070	0.060
SUV_peak_						
Difference Mean	0.354	0.391	0.634	0.734	0.530	0.315
SD	0.132	0.167	0.194	0.164	0.163	0.130
95% CI	0.037	0.046	0.054	0.045	0.045	0.036

SD = standard deviation, CI = confidence interval

### Recovery coefficient

**Fig 4:**
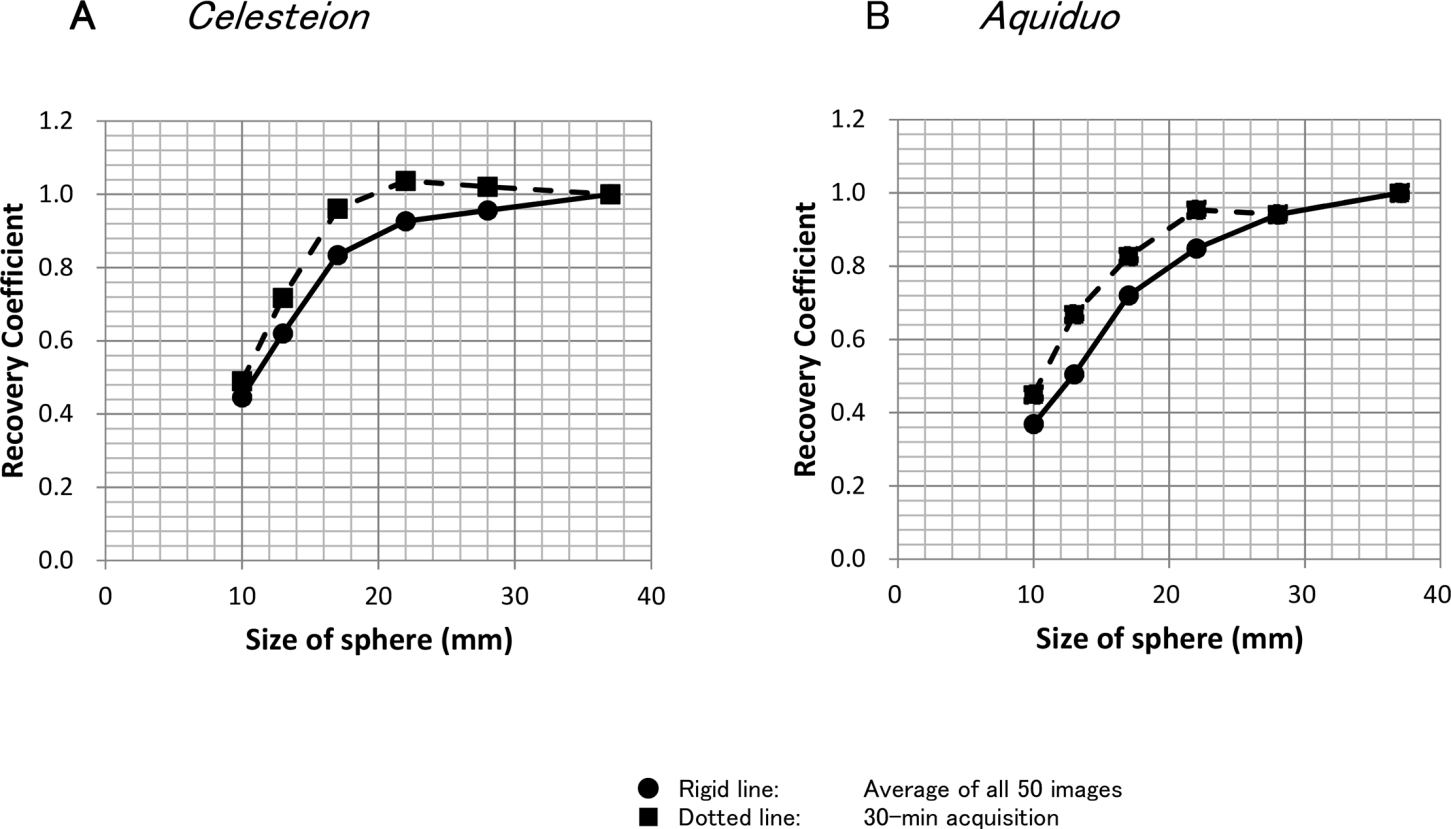
The curves of the recovery coefficients. The curves of the recovery coefficients measured by the *Celesteion* (a) and *Aquiduo* (b) scanners. The solid line with black circles corresponds to the average of the count-based measurements. The dashed line with black squares corresponds to the standard 30-min acquisition measurements.

The curves of the recovery coefficients measured by the *Celesteion* and *Aquiduo* scanners are depicted in Fig 4. The *Celesteion* scanner demonstrated higher recovery coefficients than the *Aquiduo* scanner, particularly at a diameter of approximately 20 mm. Additionally, the curves calculated from the averaged values of the count-based images exhibited lower values than those calculated from the 30-min acquisition images.

## Discussion

It is important to develop an easy and robust method to evaluate the variability and repeatability of measured SUVs, not only for daily PET examinations, but also for multi-institutional studies. Repetitive phantom scans provide valuable information regarding the characteristics of the scanner and workstation. However, as demonstrated in Fig 1, simple repetitive scans with fixed acquisition times demonstrate the tendency of the measured SUVs to increase, mainly due to the increase of noise from the decay of radioactivity [10]. To avoid the influence of radioactivity changes, the total counts for each image reconstruction should be fixed. However, the list-mode data contains information regarding the time of the counts, but not the order of the counts. Thus, it is difficult to set a fixed count threshold for each image. Thus, we developed a method to calculate the reconstruction time to maintain the counts for each image. In this study, we applied the radioactivity and acquisition times of common clinical FDG-PET studies: 2.53 kBq/cc of radioactivity at the beginning of the PET scan, and a 2-min scan for a bed position. We also operated the PET scanners under the same conditions as those of daily clinical PET examinations. To our knowledge, this is the first study that has propounded count-based image reconstruction for the evaluation of variation and repeatability of measured SUVs. We believe that our method could apply to the standardization and harmonization of SUVs in multi-institutional studies.

Our results clearly demonstrated the differences between the SUVs measured by the two different scanners. The SUVs measured by the older scanner (*Aquiduo*) were 10–30% smaller than those measured by the newer scanner (*Celesteion*). In our hospital, these scanners have been used with equal frequency. A considerable number of patients have been examined with each scanner. Such differences in SUVs may cause misinterpretation of the PET examination results. When more than one scanner is used, harmonization or calibration of SUVs is required.

Our results also suggested that the SD of the measured SUVs was between 0.1 and 0.2. There was a tendency for the smaller sphere to exhibit a larger SD, and the SUV_peak_ had a smaller SD than the SUV_max_. These results can be explained by the noise effect. The small amount of radioactivity that occurs due to the small size of the sphere increases the noise. The SUV_max_ is influenced more by the noise than the SUV_peak_, because the SUV_max_ represents the highest value in the VOI. A SD between 0.1 and 0.2 seems to be tolerable for the evaluation of clinical PET examinations, because it has been reported that tumor SUV has a within-subject coefficient of variation of approximately 10% [14]. The guidelines of clinical PET examinations and PET scanner maintenance could include the use of the SD measured with our method as an assessment criterion.

The results of the Bland-Altman analyses demonstrated poor agreement between the SUVs measured by the two scanners. The SD of the difference was several times larger than the 95% CI. We do not think that such a poor agreement was a result of unsatisfactory phantom preparation, but rather a result of the short acquisition times, corresponding to 2 min. The images obtained from such short samples have considerable noise, but comparable acquisition periods are commonly used in clinical settings. The large SD of the measured SUVs may suggest fragility of SUVs as an imaging biomarker, and should be taken into account when we interpret SUVs of a lesion.

As our results demonstrated, there were considerable differences in the recovery coefficients between the two scanners. This may be due to the newer scanner’s (*Celesteion*) better special resolution. Such technological improvements often lead to significant device-dependent and reconstruction-dependent variations in quantitative values. The European Association of Nuclear Medicine Research Ltd. (EARL) accreditation program [15], the North American QIBA, and the Uniform Protocols in Clinical Trials (UPICT) [16] all recommend that adequate reproducibility of SUVs be achieved by patient preparation, as well as acquisition and reconstruction parameters. It was recently shown that it is possible to harmonize SUVs by applying the Gaussian filters [17]. This method requires the recovery coefficients calculated from the images of a NEMA IEC body phantom. However, such phantom images have been generally obtained with much longer (10–30 min) acquisition times than that of a daily oncology PET examination (approximately 2 min/bed). As shown in Fig 4, the differences in the acquisition times result in differences in the recovery coefficients. From this perspective, we suggest the use of the averaged values obtained from count-based image reconstruction, under the same conditions as actual clinical settings. Using such values obtained from another scanner, we can harmonize SUVs between the two scanners. For example, our results showed that an SUV of 4.0 for a 22-mm-diameter sphere was measured as an SUV_max_ of 4.7 by the Celesteion and of 3.8 by the Aquiduo. We can harmonize the SUV_max_ of 4.7 obtained by the Celesteion with the value of 3.8 obtained by the Aquiduo, or the other way around. Alternatively, SUV_max_ values from both the Celesteion and Aquiduo could be calibrated to the actual SUV of 4.0, which can be called as a standardization. The measured SUVs depend mainly on the size of lesion and the actual SUV [10]. Thus, it is necessary to evaluate a phantom that is set for several SUVs. Correction for partial volume effects might also be useful for small lesions [18]. Validation of these methods using clinical data is required, and this will be a subject for further study.

There is a limitation of our count-base method. Some of the trend lines of SUVs in Fig 2 show a slight upward trend. This may suggest that our method is limited in its ability to correct the counts in each reconstruction. Our method cannot exclude the influence of random coincidences, statistical noise, or dead-time losses. The noise equivalent count rate of the scanner may be useful for further correction.

## Conclusions

We propose the use of count-based image reconstruction to obtain a considerable number of images under clinical settings from hours of list-mode scanning. These results are useful for evaluating the variability and repeatability of measured SUVs. The results for various scanners are also useful for standardization and harmonization of SUVs in multi-institutional studies.
